# Combined Antitumor Therapy Using In Situ Injectable Hydrogels Formulated with Albumin Nanoparticles Containing Indocyanine Green, Chlorin e6, and Perfluorocarbon in Hypoxic Tumors

**DOI:** 10.3390/pharmaceutics14010148

**Published:** 2022-01-08

**Authors:** Woo Tak Lee, Johyun Yoon, Sung Soo Kim, Hanju Kim, Nguyen Thi Nguyen, Xuan Thien Le, Eun Seong Lee, Kyung Taek Oh, Han-Gon Choi, Yu Seok Youn

**Affiliations:** 1 School of Pharmacy, Sungkyunkwan University, 2066 Seobu-ro, Jangan-gu, Suwon 16419, Gyeonggi-do, Korea; wtak94@naver.com (W.T.L.); morningbright96@gmail.com (J.Y.); alsajls417@gmail.com (S.S.K.); gkswn4564@naver.com (H.K.); ngtnguyen1710@gmail.com (N.T.N.); lexuanthien.dkh@gmail.com (X.T.L.); 2 Department of Biotechnology and Department of Biomedical-Chemical Engineering, The Catholic University of Korea, 43 Jibong-ro, Bucheon-si 14662, Gyeonggi-do, Korea; eslee@catholic.ac.kr; 3 College of Pharmacy, Chung-Ang University, 84 Heukseok-ro, Dongjak-gu, Seoul 06974, Korea; kyungoh@cau.ac.kr; 4 College of Pharmacy, Hanyang University, 55 Hanyangdaehak-ro, Sangnok-gu, Ansan 15588, Gyeonggi-do, Korea; hangon@hanyang.ac.kr

**Keywords:** photothermal therapy, photodynamic therapy, combined antitumor effect, oxygenation, hydrogel, hypoxic tumor

## Abstract

Combined therapy using photothermal and photodynamic treatments together with chemotherapeutic agents is considered one of the most synergistic treatment protocols to ablate hypoxic tumors. Herein, we sought to fabricate an in situ-injectable PEG hydrogel system having such multifunctional effects. This PEG hydrogel was prepared with (i) nab^TM^-technique-based paclitaxel (PTX)-bound albumin nanoparticles with chlorin-e6 (Ce6)-conjugated bovine serum albumin (BSA-Ce6) and indocyanine green (ICG), named ICG/PTX/BSA-Ce6-NPs (~175 nm), and (ii) an albumin-stabilized perfluorocarbon (PFC) nano-emulsion (BSA-PFC-NEs; ~320 nm). This multifunctional PEG hydrogel induced moderate and severe hyperthermia (41−42 °C and >48 °C, respectively) at the target site under two different 808 nm laser irradiation protocols, and also induced efficient singlet oxygen (^1^O_2_) generation under 660 nm laser irradiation supplemented by oxygen produced by ultrasound-triggered PFC. Due to such multifunctionality, our PEG hydrogel formula displayed significantly enhanced killing of three-dimensional 4T1 cell spheroids and also suppressed the growth of xenografted 4T1 cell tumors in mice (tumor volume: 47.7 ± 11.6 and 63.4 ± 13.0 mm^3^ for photothermal and photodynamic treatment, respectively, vs. PBS group (805.9 ± 138.5 mm^3^), presumably based on sufficient generation of moderate heat as well as ^1^O_2_/O_2_ even under hypoxic conditions. Our PEG hydrogel formula also showed excellent hyperthermal efficacy (>50 °C), ablating the 4T1 tumors when the irradiation duration was extended and output intensity was increased. We expect that our multifunctional PEG hydrogel formula will become a prototype for ablation of otherwise poorly responsive hypoxic tumors.

## 1. Introduction

Photodynamic therapy (PDT) has become a prominent therapeutic modality for cancer treatment [1,2,3]. Briefly, PDT is based on the clinical use of reactive oxygen species (ROS) such as singlet oxygen (^1^O_2_) or hydroxyl radicals (OH) generated from oxygen (O_2_) using photosensitizers (PS) and light energy. The intracellularly generated ROS are able to damage cancer cells and tumor vasculature and also induce immune-mediated tumor suppression [3]. To date, several PDT agents, such as Photofrin^®^, Foscan^®^, and Visudyne^®,^ etc., have been approved by the U.S. FDA, and the therapeutic availability of PDT has expanded toward cancer therapy. However, the clinical application of PDT is often restricted because most PS agents are effective only under focused laser illumination (650–900 nm) in the presence of sufficient oxygen. Unlike normal tissues, oxygenation in primary tumors is not regulated, and thus the partial oxygen pressure of tumors is often heterogeneous and can be remarkably low [4]. Interestingly, the O_2_ concentration within human tumors is known to be 1.3%~3.9% (mostly less than 0.33%), in comparison with 3.1%~8.7% within normal tissues [4,5]. The efficacy of many PDT and chemotherapeutic agents is seriously impaired by low oxygen concentrations within hypoxic tumors, which causes hypoxia-induced resistance of tumors to antitumor therapy [6]. Therefore, increasing the oxygen concentration in hypoxic tumors can be an effective method to overcome hypoxia-induced resistance of tumors to PDT [7,8,9].

Photothermal therapy (PTT) is also a promising antitumor modality that acts by raising tissue temperature [10]. Cancer cells are susceptible to temperature elevation and are often killed as a result of irreversible damage and subsequent induction of apoptosis even after exposure to temperatures >48 °C for only 5 min [11]. Many organic PTT agents emit heat in response to focused near-infrared (NIR) laser irradiation at wavelengths of 650−900 nm [10,12]. Because the hyperthermia can be concentrated within the area targeted by the NIR irradiation, PTT may create relatively little damage to surrounding healthy tissues [10,13]. Moreover, PTT can also be used to induce moderate local hyperthermia (39–42 °C) that promotes the delivery of oxygen as well as chemotherapeutics into tumor tissues due to elevated tumor blood flow [14,15]. This moderate PTT is useful in clinical settings because it attenuates the tumor hypoxic state and improves the efficacy of chemotherapeutic agents [16,17].

Albumin is a versatile carrier due to its excellent pharmaceutical advantages, such as biodegradability, biocompatibility, high stability, and solubility [18,19]. It has been used as a building material to fabricate a great number of therapeutic and diagnostic drug delivery systems, including nanoconjugates [20,21], nano-/micro-particles [22,23,24,25], and hydrogels of various sizes [26,27,28,29]. In practice, the nab^TM^ (nanoparticle albumin-bound)-based paclitaxel formulation Abraxane^®^ (Celgene Corp.) has displayed enhanced antitumor efficacy over the conventional paclitaxel formulation of Taxol^®^. In particular, this prototypical nab^TM^ formula has sufficient flexibility and expandability to entrap various hydrophobic drugs, is better able to target the tumor gp60-mediated transcytosis pathway [18], and has been extensively used as a biocompatible component of injectable hydrogels [26,27,28,29].

In this study, we developed a multifunctional in situ-forming PEG-albumin hydrogel that was able to generate oxygen (O_2_) and singlet oxygen (^1^O_2_) and emit moderate or severe heat for the combination treatment of hypoxic tumors using chemotherapeutic, photodynamic, and photothermal therapy. This PEG hydrogel formula was designed to include (i) albumin nanoparticles containing paclitaxel (PTX), chlorin e6 (Ce6), and indocyanine green (ICG), and (ii) an albumin-stabilized perfluorocarbon (PFC) nano-emulsion (NE). The capability of this PEG hydrogel formula to generate heat and oxygen/singlet oxygen was carefully evaluated. The physicochemical and photo-induced properties of this multifunctional PEG hydrogel were also assessed by using relevant analyses. Finally, the combined chemotherapeutic, photodynamic and photothermal effects of this PEG hydrogel formula were carefully evaluated in 4T1 cell spheroids under hypoxic conditions and in 4T1 tumor-bearing mice.

## 2. Materials and Methods

### 2.1. Materials

Paclitaxel (PTX) was obtained from JW Pharmaceutical Corporation (Dangjin, South Korea). Indocyanine green (ICG) and icosafluoro-15-crown-5-ether (PFC) were purchased from Tokyo Chemical Industry Co., LTD. (Tokyo, Japan). Chlorin e6 (Ce6) was supplied by Frontier Scientific (Salt Lake City, UT, USA). Bovine serum albumin (BSA) and 2-iminothiolane (2-IT; Traut’s reagent) were purchased from Sigma-Aldrich (St. Louis, MO, USA). Four-arm polyethylene glycol maleimide (4-arm PEG-MAL; Mw 20 kDa) was purchased from NOF Corporation (Tokyo, Japan). The 4T1 breast cancer cells were obtained from the American Type Culture Collection (ATCC; Rockville, MD, USA). LIVE/DEAD™ viability/cytotoxicity assay kits, Singlet Oxygen Sensor Green reagent (SOSG), and CellROX™ Deep Red reagent were purchased from Thermo Fisher Scientific (Waltham, MA, USA). Anti-HIF-1α primary and Alexa Fluor^®^-488-conjugated goat anti-rabbit secondary antibodies were purchased from Abcam (Cambridge, MA, USA). Trypsin-EDTA and penicillin-streptomycin (P/S) solution were purchased from Corning (Somerville, NY, USA). Dulbecco’s Modified Eagle Medium (DMEM) and fetal bovine serum (FBS) were purchased from Capricorn (Ebsdorfergrund, Hesse, Germany). All other reagents were obtained from Sigma-Aldrich unless otherwise indicated.

### 2.2. Animals

The animals were cared for in accordance with the Guide for the Care and Use of Laboratory Animals published by the United States National Institutes of Health. The protocols were also approved by the Institutional Animal Care and Use Committee (IACUC) of Sungkyunkwan University (IACUC number: 202106291, approval date 15 July 2021). The BALB/c *nu*/*nu* mice (female, 6 weeks old) were purchased from ORIENT BIO (Seongnam, South Korea). Mice were housed in microisolator cages on individually ventilated cage racks with ad libitum access to an autoclaved standard rodent diet (LabDiet 5008, Purina, St. Louis, MO, USA) and were kept under a 12 h light/dark cycle.

### 2.3. Synthesis of Ce6-Conjugated BSA (BSA-Ce6)

The BSA was covalently modified with Ce6 using a slight modification of the previous method [2]. Briefly, Ce6 (1 mmol), N-dicyclohexylcarbodiimide (DCC, 4 mmol), and N-hydroxysuccinimide (NHS, 5 mmol) were dissolved in 10 mL anhydrous dimethyl sulfoxide (DMSO) in a glass tube. Triethylamine (TEA, 4 mmol) was added to the mixture, and the reaction was performed in the dark at ambient temperature for 24 h. After removing the precipitate, the NHS-activated Ce6 was stored at −70 °C until required. Separately, 1.5 mL of Ce6-NHS (150 μmol) in DMSO was mixed dropwise with 50 mL of BSA (7.5 μmol; 0.1 M sodium borate buffer, pH 8.5) at a feeding ratio of 20:1 (Ce6:BSA). The reaction was allowed to continue at 450 rpm in the dark condition for 24 h. Unreacted Ce6 and DMSO were removed by using a dialysis membrane (MwCO: 10 kDa; Spectrum Labs, Rancho Dominguez, CA, USA) against 60% ethanol and deionized water (DW) for 48 and 24 h, respectively 24 h. Finally, Ce6-BSA was concentrated in DW using a centrifugal concentrator (MwCO: 30 kDa, Amicon^®^ Ultra, Millipore, Burlington, MA, USA) and then lyophilized and stored at −20 °C for further use.

### 2.4. Preparation of ICG/PTX/BSA-Ce6-NPs

The ICG/PTX/BSA-Ce6-NPs were prepared as previously described using the nanoparticle albumin-bound (nab^TM^) technology with some adjustments [2,30,31,32,33,34]. In brief, 45 mg BSA, 5 mg BSA-Ce6, and 0.75 mg ICG were dissolved in 5 mL DW. Then, 5 mg PTX was dissolved in 0.1 mL of a 9:1 solution (chloroform:ethanol). These two solutions were gently shaken and homogenized by using a high-speed homogenizer (WiseTis^®^ HG-15D: DAIHAN Scientific Co., Seoul, South Korea) at 14,500 rpm for 3 min, and then passed through a high-pressure homogenizer (EmulsiFlex-B15 device, Avestin, Ottawa, ON, Canada) for nine cycles at 20,000 psi. The resulting dispersion was rotary evaporated for 15 min under reduced pressure at 40 °C to remove chloroform and ethanol. The nanoparticle fraction was centrifuged at 6000 rpm, and the supernatant was collected. The resulting suspension was lyophilized and stored at −20 °C until required.

### 2.5. Fabrication of BSA-Stabilized PFC Nano-Emulsion

The BSA-PFC nano-emulsion (BSA-PFC-NEs) was fabricated using a slight modification of the previous method [35]. A 300 μL aliquot of icosafluoro-15-crown-5-ether (PFC) was mixed with 4 mL of PBS containing 40 mg of BSA. The mixture was held in an ice bath and ultrasonicated (operation 8 s, interval 2 s) at an amplitude of 20% for 400 s. The resulting nano-emulsion was centrifuged (8000 rpm, 3 min), and the resulting nano-emulsion pellets were re-dispersed in 0.1 mL PBS.

### 2.6. Characterization of ICG/PTX/BSA-Ce6-NPs and BSA-PFC-NEs

The particle size and zeta potential of ICG/PTX/BSA-Ce6-NPs and BSA-PFC-NEs (DW) were measured by using a Zetasizer Nano ZS90 instrument (Malvern Instruments, Worcestershire, U.K.) at a dynamic light scattering mode. The surface morphology of the NPs was observed by transmission electron microscopy (TEM: JEM-3010, JEOL, Tokyo, Japan) and field-emission scanning electron microscopy (FE-SEM: JSM7000F, JEOL, Tokyo, Japan). The particle sizes of ICG/PTX/BSA-Ce6-NPs and BSA-PFC-NEs were measured at 0, 1, 2, 4, 8, 12, 24, 48, and 72 h at room temperature. In addition, UV-VIS-NIR spectral scans of ICG/PTX/BSA-Ce6-NPs or free ICG/Ce6 were also recorded over a range of wavelengths (300–900 nm) using a Synergy™ NEO microplate reader (Bio Tek, Winooski, VT, USA).

### 2.7. Preparation of In Situ-Gelling PEG Hydrogel with ICG/PTX/BSA-Ce6-NPs and BSA-PFC-NEs

The PEG hydrogel with ICG/PTX/BSA-Ce6-NPs and BSA-PFC-NEs (ICG/PTX/BSA-Ce6-NPs~PFC-NEs@Gel) was formulated using an in situ-gelling PEG hydrogel according to the protocol described previously with modifications [26,27,36]. To synthesize thiolated BSA (BSA-SH), BSA (500 mg) was dissolved in 1 mL of 100 mM PBS buffer (pH 8.0) containing 2-iminothiolane (2-IT, 12.5 mg), and the mixture was allowed to react by stirring for 2.5 h. The unreacted 2-IT was removed using a centrifugal concentrator (MwCO: 10 kDa, Amicon^®^ Ultra, Millipore). The formula (1 mL) of the in situ-gelling PEG hydrogel comprised (i) ICG/PTX/BSA-Ce6-NPs (300 µL; 30 mg/mL), (ii) BSA-PFC-NEs (100 µL as a final emulsion), (iii) BSA-SH (150 µL; 500 mg/mL), and (iv) 4-arm PEG-MAL (450 µL; 11.4 mg/mL). Each component was mixed immediately, and gelation was optimized by monitoring the gelation time according to the molar ratio of 4-arm PEG-MAL and BSA-SH (BSA-SH:4-arm PEG-MAL = 1:3.3~4.5). The material was considered to be in a gel state if it did not flow when inverted.

### 2.8. Characterization of ICG/PTX/BSA-Ce6-NPs~PFC-NEs@Gel

The different PEG hydrogel formulas (Z1~Z6) with ICG/PTX/BSA-Ce6-NPs and BSA-PFC-NEs were designated as follows: (i) Z1 = PEG hydrogel; (ii) Z2 = PTX/BSA-NPs@Gel; (iii) Z3 = BSA-PFC-NEs@Gel; (iv) Z4 = PTX/BSA-Ce6-NPs@Gel; (v) Z5 = ICG/PTX-BSA-NPs@Gel; (vi) Z6 = ICG/PTX/BSA-Ce6-NPs~PFC-NEs@Gel (Table 1). General features of the hydrogel were characterized using photographs, photothermal images (FLIR E85 photothermal camera, FLIR Systems, Inc., Wilsonville, OR, USA), and fluorescence images (FOBI in vivo imaging system, NeoScience, Suwon, Korea).

### 2.9. In Vitro and In Vivo Photothermal Imaging and Hyperthermia Monitoring

A thermal imaging camera was used to measure the photothermal effect of the ICG/PTX/BSA-Ce6-NPs in vitro and in vivo using a slight modification of the previous method [26]. Naïve PEG hydrogel or ICG/PTX/BSA-Ce6-NPs@Gel in 1.5 mL Eppendorf tubes was irradiated for 5 min with an 808-nm laser (0.8 or 1.2 W/cm^2^ to induce mild or severe hyperthermia, respectively). To obtain photothermal images in vivo, naive PEG hydrogel or ICG/PTX/BSA-Ce6-NPs@Gel was injected into the tumors of mice, and the tumor regions were either irradiated or not irradiated with 808 nm laser (0.6–0.8 W/cm^2^) for 20 min. Temperature changes within the tumor areas were recorded and observed using a FLIR E85 photothermal camera (FLIR Systems, Inc., Wilsonville, USA).

### 2.10. Singlet Oxygen Generation by ICG/PTX/BSA-Ce6-NPs

Singlet Oxygen Sensor Green (SOSG) reagent was used to assess singlet oxygen generation from ICG/PTX/BSA-Ce6-NPs. Free Ce6 and ICG/PTX/BSA-Ce6-NPs were mixed with the SOSG reagent (final concentration 10 μM) in a 24-well plate. A 660-nm laser (10 mW/cm^2^) irradiated the respective sample wells for free Ce6 and ICG/PTX/BSA-Ce6-NPs under either normoxic or hypoxic conditions. Hypoxia was precisely achieved by incubating the hypoxic chamber in a hypoxic gas stream (5% CO_2_:95% N_2_ on a volume basis). While irradiating the sample wells with the laser for 60 min, a 100 μL aliquot of each sample was collected every 10 min. The intensity of the green fluorescence emitted from SOSG induced by ^1^O_2_ generation was determined using a multi-mode microplate reader (excitation 485 nm; emission 538 nm).

### 2.11. Encapsulation Efficiency and Release Profiles of PTX, ICG, and BSA-Ce6

The encapsulation efficiency (EE) of PTX, BSA-Ce6, and ICG into ICG/PTX/BSA-Ce6-NPs was examined. Briefly, 5 mg of the lyophilized ICG/PTX/BSA-Ce6-NPs was dissolved in 0.5 mL DW, and then 4.5 mL acetonitrile (ACN) was added to the NPs solution, followed by sonication for 30 min and centrifugation for 15 min (14,500 rpm). Subsequently, the supernatant was withdrawn for the quantification of PTX and ICG. The EE of BSA-Ce6 was calculated by measuring the supernatant concentration of BSA-Ce6 after the nanoparticle preparation. For the release test, a 5 mL ICG/PTX/BSA-Ce6-NPs solution (10 mM PBS, pH 7.4) was loaded into the PEG hydrogel. The drug release from the hydrogel was carried out using a gently rotated bench-top rotator (150 rpm, 37 °C). At predetermined times, a 0.15 mL aliquot was withdrawn and centrifuged (14,500 rpm, 10 min), and the fluorescence intensities of the supernatants were measured by using a fluorescence spectrometer (FluoroMax Plus; HORIBA Scientific, Tokyo, Japan). Three replicates of each sample were prepared and analyzed (n = 3). The excitation/emission wavelengths used for ICG and Ce6 were 420/526 and 405/670 nm, respectively. To determine the loading efficiency of PTX, the supernatant was subjected to reverse-phase high-performance liquid chromatography (RP-HPLC) using a PLRP-S Zorbax 100 RP-18 column (150 × 4.6 mm, 8 µm/300 Å; Agilent Technologies, Palo Alto, CA, USA). An isocratic elution method was performed at a flow rate of 1.0 mL/min using a mixed solution (DW:ACN = 45:55). Eluates were monitored at 220 nm. Drug loading efficiency (%) was calculated as amount of drug entrapped/amount of drug loaded × 100.

### 2.12. Cytotoxicity of ICG/PTX/BSA-Ce6-NPs in 4T1 Cell Spheroids Using LIVE/DEAD^TM^ Cell Assay

Three-dimensional culture of 4T1 cells to form spheroids was performed by a slight modification of previously described methods [2,26,30]. LIVE/DEAD™ viability/cytotoxicity kits were used to visualize the death and viability of the 4T1 cells. Briefly, the 4T1 cells were cultured in DMEM media containing 10% (*v*/*v*) fetal bovine serum and 1% penicillin/streptomycin in a 5% CO_2_, 95% RH incubator at 37 °C. A 100 μL sample containing 4000 cells was seeded into each well of V-bottom cell plates (Shimadzu, Kyoto, Japan) designed to induce the formation of 3D multicellular spheroids. The 4T1 cell spheroids were then allowed to become established over three days before further experiments. The spheroids previously incubated with ICG/PTX/BSA-Ce6-NPs@Gel for 6 h were assigned to groups with or without laser irradiation (808 nm for 10 min or 660 nm for 60 min, respectively) and/or with or without an ultrasound (US) scan (US for 15 min) according to the previously described oxygen conditions (normoxic, hypoxic or oxygenated). The degree of hyperthermia induced was determined irradiation at 0.8 and 1.2 W/cm^2^ for mild and severe hyperthermia, respectively, where the target temperatures were set to 41–42 °C or >48 °C, respectively. The hypoxic condition was precisely achieved by incubating the hypoxic chamber in a hypoxic gas stream (5% CO_2_:95% N_2_ on a volume basis). The plates for samples were washed three times with DPBS, and the medium was replenished with fresh medium before laser or ultrasound treatment. After laser or ultrasound treatment, the cells were stained with Calcein-AM (for live cells) and Ethidium Homodimer-1 (for dead cells). Subsequently, the stained cells were observed by CLSM (excitation/emission wavelengths of 494/517 nm for Calcein-AM and 528/617 nm for Ethidium Homodimer-1, respectively).

### 2.13. ROS Deep Assay at 4T1 Cell Spheroids

The 4T1 cell spheroids were plated into each well of an 8-well chamber (Ibidi, Martinsried, Germany) and incubated with ICG/PTX/BSA-Ce6-NPs for 6 h. Cell spheroids were replenished with fresh DMEM (1% FBS) prior to laser irradiation (660 nm) and a US scan performed under normoxic, hypoxic, or oxygenated conditions. CellROX^®^ Reagent was added to the cell spheroids at a final concentration of 20 µM and the plates were incubated at 37 °C for 2 h. Spheroids were washed three times with DPBS and stained with DAPI. The intracellular distribution of ROS in the spheroid cells was visualized using confocal laser scanning microscopy (CLSM; Carl Zeiss, Jena, Germany).

### 2.14. HIF-1α Visualization at 4T1 Cell Spheroids

The 4T1 cell spheroids were plated into each well of an 8-well chamber (Ibidi, Martinsried, Germany) and incubated with BSA-PFC-NEs for 1 h. The cell spheroids were subjected to a US scan under normoxic, hypoxic, or oxygenated conditions. Hypoxic conditions were achieved by incubating the hypoxic chamber in a hypoxic gas stream (5% CO_2_:95% N_2_ on a volumetric basis). The spheroids were fixed with 4% formaldehyde for 60 min and incubated for 90 min with 0.1% Triton X- 100, and then incubated with 1% BSA, 22.52 mg/mL glycine in PBST (PBS + 0.1% Tween 20) for 2 h to block unspecific binding of the antibodies. The resulting spheroids were further incubated with the diluted antibody solution (anti-HIF-1 alpha antibody-Alexa Fluor 488; 1/25) in 1% BSA in PBST in a humidified chamber for 1 h at room temperature. After removing the supernatant, the cell spheroids were washed three times in PBS, incubated with DAPI (DNA stain) for 4 h, and further rinsed with PBS. Samples were observed using CSLM.

### 2.15. In Vivo Imaging of ICG/PTX/BSA-Ce6-NPs

The in situ-gelling ICG/PTX/BSA-Ce6-NPs~PFC-NEs@Gel (100 μL) were directly injected into 4T1-cell-xenografted tumors on mice. At predetermined times, the fluorescence signals of Ce6 and ICG originating from the tumors were visualized at 630 and 730 nm, respectively, and at predetermined times using a FOBI in vivo imaging system (NeoScience, Suwon, Korea).

### 2.16. In Vivo Photoacoustic Imaging

Photoacoustic (PA) imaging was used to determine the saturation/distribution of oxygen or PFC gas inside the xenografted 4T1 tumors. When the tumors reached ~150 mm^3^ volumes, the mice were treated with (i) PBS; (ii) ICG/PTX/BSA-Ce6-NPs~PFC-NEs@Gel + US(+); (iii) ICG/PTX/BSA-Ce6-NPs~PFC-NEs@Gel + US(−). At before and after (30 min) intratumor injections, tumor oxygenation (as the oxyhemoglobin:HbO_2_ ratio) was visualized and recorded using a Vevo^®^ LAZR-X Multimodal Imaging System (FujiFilm, VisualSonics Inc., Minato, Tokyo, Japan) set to the Oxy-hem mode (750 and 850 nm) at predetermined times.

### 2.17. In Vivo Antitumor Efficacy in 4T1 Cell-Xenograft Mice

The 4T1 cells (100 μL; 3 × 10^6^ cells) were injected subcutaneously into the dorsal flanks of the mice. When the volume of the tumors reached ~150 mm^3^, the mice were randomly divided into five groups (G1−G5) as follows: (I) control: PBS; (II) chemotherapeutic = PTX/BSA-NPs@Gel (100 μg PTX (0.1 mL/mouse); (III) deoxy-photodynamic = ICG/PTX/BSA-Ce6-NPs@Gel + 660 nm (+); (IV) severe hyperthermia = ICG/PTX/BSA-Ce6-NPs@Gel + 808 nm(+); and (V) oxy-photodynamic plus moderate hyperthermia = ICG/PTX/BSA-Ce6-NPs~PFC-NEs@Gel + 660 nm (+) + 808 nm(+) + US(+) (Table 1). All treatments were injected intratumorally. Tumor volume and body weight of the mice were measured every day. Tumor volume (V) was calculated using the equation V = 0.5 × A × B^2^, where A is length (longest diameter) and B is width (shortest diameter). On day 14, the mice were sacrificed, and their tumors were harvested and photographed. The tumors were stored in formalin for further experiment. 

### 2.18. Data Analyses

All data are presented as the mean ± standard deviation (SD). The significance of differences between treatment group results was determined using Student’s *t*-test, and *p*-values < 0.05 were considered statistically significant.

## 3. Results

### 3.1. Preparation and Characterization of ICG/PTX/BSA-Ce6-NPs and BSA-PFC-NEs

**Figure 1:**
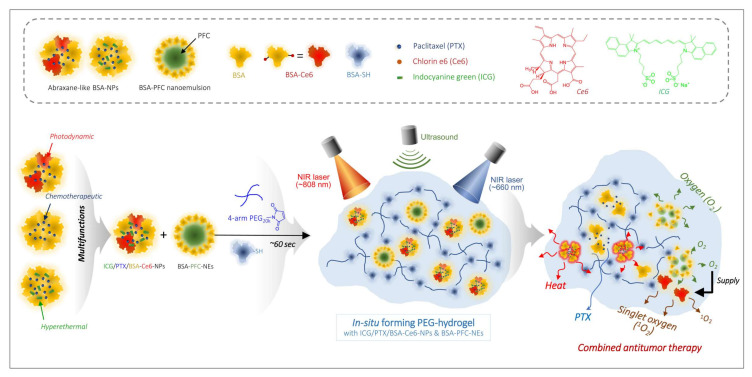
Schematic illustration of combined photothermal and photodynamic therapy for hypoxic tumors using ICG/PTX/BSA-Ce6-NPs~PFC-NEs@Gel under laser irradiation (660/808 nm) and ultrasound trigger.

**Figure 2:**
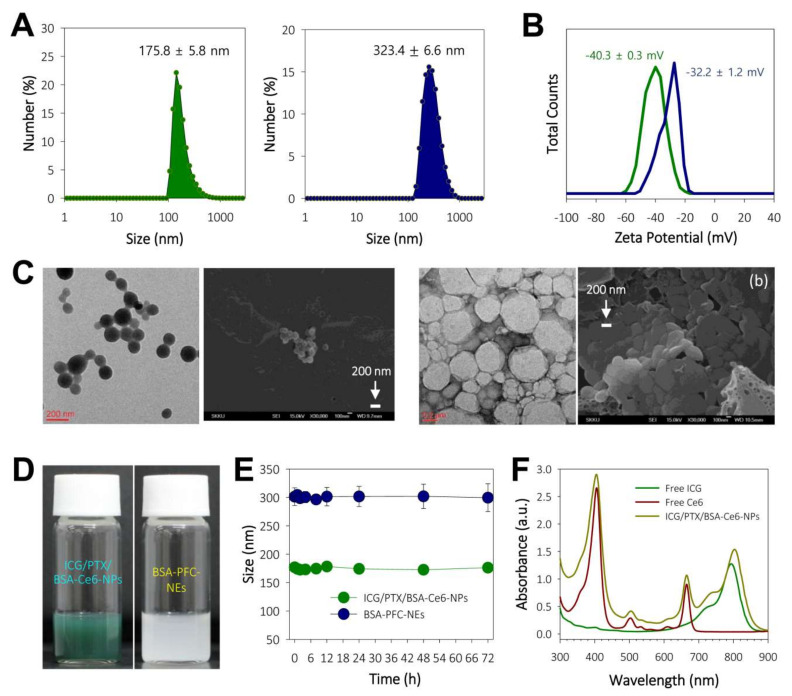
(**A**) Histograms of particle sizes of ICG/PTX/BSA-Ce6-NPs and BSA-PFC-Nes. (**B**) Zeta potentials of ICG/PTX/BSA-Ce6-NPs and BSA-PFC-NEs. (**C**) TEM and FE-SEM images of ICG/PTX/BSA-Ce6-NPs (left: a) and BSA-PFC-NEs (right: b). (**D**) Photographs showing colloidal solution state for ICG/PTX/BSA-Ce6-NPs and BSA-PFC-NEs. (**E**) Physical stability of ICG/PTX/BSA-Ce6-NPs and BSA-PFC-NEs based on size changes over 3 days. (**F**) UV-VIS spectroscopic analyses for free ICG, free Ce6, and ICG/PTX/BSA-Ce6-NPs at 300–900 nm wavelengths.

The ICG/PTX/BSA-Ce6-NPs and BSA-PFC-NEs were fabricated using a high-pressure homogenizer based on the nab^TM^ technology and ultrasonication, respectively (Figure 1). The sizes of the prepared ICG/PTX/BSA-Ce6-NPs and BSA-PFC-NEs were 175.8 ± 5.8 and 323.4 ± 6.6 nm, respectively (Figure 2A). Their zeta potentials were shown to be −40.3 ± 0.3 and −32.2 ± 1.2, respectively (Figure 2B). The ICG/PTX/BSA-Ce6-NPs were highly homogeneous and spherical, whereas the BSA-PFC-NEs appeared to be rather irregular and less homogeneous (Figure 2C). These solutions of ICG/PTX/BSA-Ce6-NPs and BSA-PFC-NEs showed the typical characteristics of a colloid or nano-emulsion (Figure 2D). In particular, the particle sizes of both the ICG/PTX/BSA-Ce6-NPs and BSA-PFC-NEs remained stable over a three-day evaluation period (Figure 2E). When observed at wavelengths ranging between 300 and 900 nm, the ICG/PTX/BSA-Ce6-NPs were found to have typical absorption profiles of indocyanine green and chlorin e6 of around ~800 nm and 660 nm, respectively. The Ce6-BSA, BSA-BC-NPs, and Ce6-BSA-BC-NPs were investigated (Figure 2F). In addition, the loading efficiency of PTX, ICG, and BSA-Ce6 was found to be ~73.0%, 53.2%, and 82.1%, respectively.

### 3.2. Preparation, Characterization, and Drug Release Profiles of ICG/PTX/BSA-Ce6-NPs~PFC-NEs@Gel

**Figure 3:**
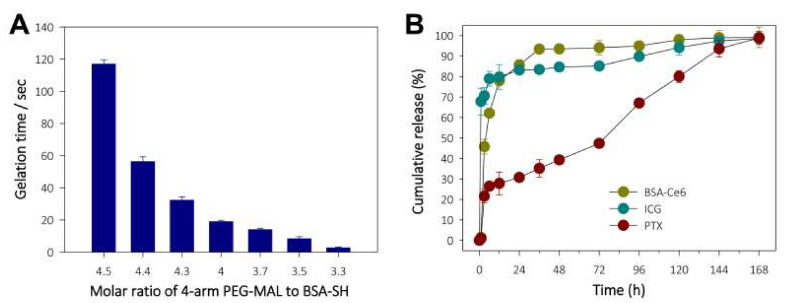
(**A**) Monitoring of gelation time for in situ-forming PEG hydrogel according to the 4-arm PEG-MAL:BSA-SH molar ratio. (**B**) Release profiles for BSA-Ce6, ICG, and PTX from ICG/PTX/BSA-Ce6-NPs@Gel.

Given that the gelation process depends on a rapid thiol-maleimide reaction, in situ gelling of the PEG hydrogel was evaluated by monitoring the relationship between the gelation time and the molar ratio of 4-arm PEG-MAL to BSA-SH. As shown in Figure 3A, reducing the 4-arm PEG-MAL to BSA-SH molar ratio sharply reduced the gelation time, with the PEG hydrogel becoming solid in approximately 1 min at a 4-arm PEG-MAL to BSA-SH ratio of 4.4:1.0, which was considered optimal to perform a series of related steps (i.e., material mixing, syringe filling, and injection). The in vitro release profiles of Ce6 (as a BSA-Ce6 conjugate), ICG, and PTX from ICG/PTX/BSA-Ce6-NPs~PFC-NEs@Gel were observed over 168 h. As shown in Figure 3B, >90% amount of BSA-Ce6 and ICG was released over 24 h with initial burst-out, and the remaining amount of both BSA-Ce6 and ICG appeared to be completely released from the PEG hydrogel after 144 h, which was appropriate for the in vivo laser irradiation protocol. In contrast, PTX displayed a gradual linear release pattern consisting without significant initial fast release until 168 h, which was presumably due to the hydrophobic property, tight binding to albumin molecules, and low solubility/diffusion of PTX. Thus, PTX seemed to have considerable time to be released from the PEG hydrogel. Subsequently, the hydrophobicity difference between PTX, ICG, and BSA-Ce6 led to a great discrepancy in the release pattern.

**Figure 4:**
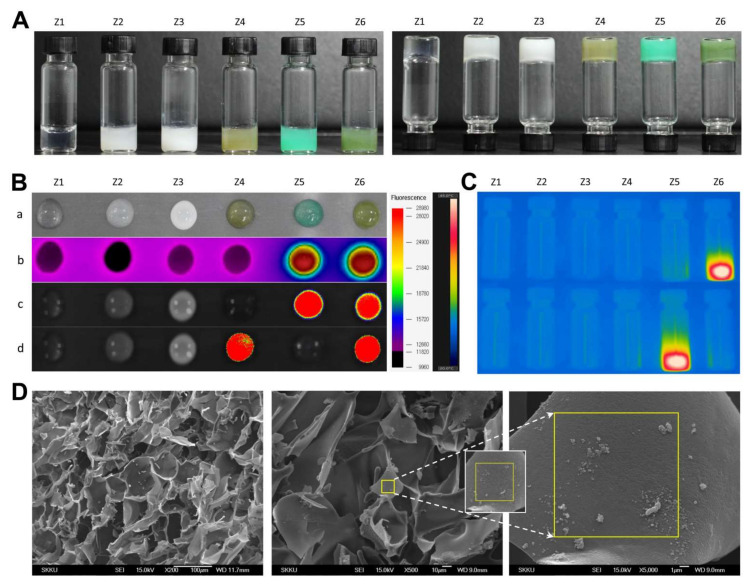
(**A**) Photographs of PEG hydrogel formulation groups (Z1–Z6) in the regular (left) and upside-down (right) state. (**B**) Various images of PEG hydrogel formulation groups (Z1–Z6) (a) photograph at 10 min after gelation, (b) thermal images, (c) fluorescence images of ICG, (d) fluorescence images of Ce6. (**C**) Thermal images of glass vials containing the Z1−Z6 PEG hydrogel formulations during laser irradiation at 420/526 and 405/670 nm, excitation/emission wavelengths to identify ICG and Ce6, respectively. (**D**) FE-SEM images for the morphology of inner structures of ICG/PTX/BSA-Ce6-NPs@Gel.

Separately, the various hydrogel formulations (Z1−Z6) had distinct colors, ranging from transparent to milky, khaki green, or dark green, depending on ICG, Ce6, and PFC concentrations (Figure 4A). All formulas gelled easily within ~60 s and did not flow or drop when turned upside-down. As shown in Figure 4B,C, PEG hydrogels containing ICG (Z5 and Z6) produced significant hyperthermia, and those containing both ICG and Ce6 emitted strong fluorescence at the relevant emission wavelengths. The FE-SEM images showed that the PEG hydrogels contained considerable amounts of nanoparticles having sizes of ~150 and >300 nm, presumably corresponding to ICG/PTX/BSA-Ce6-NPs and BSA-PFC-NEs (Figure 4D).

### 3.3. Photothermal and Photodynamic Activity of ICG/PTX/BSA-Ce6-NPs

**Figure 5:**
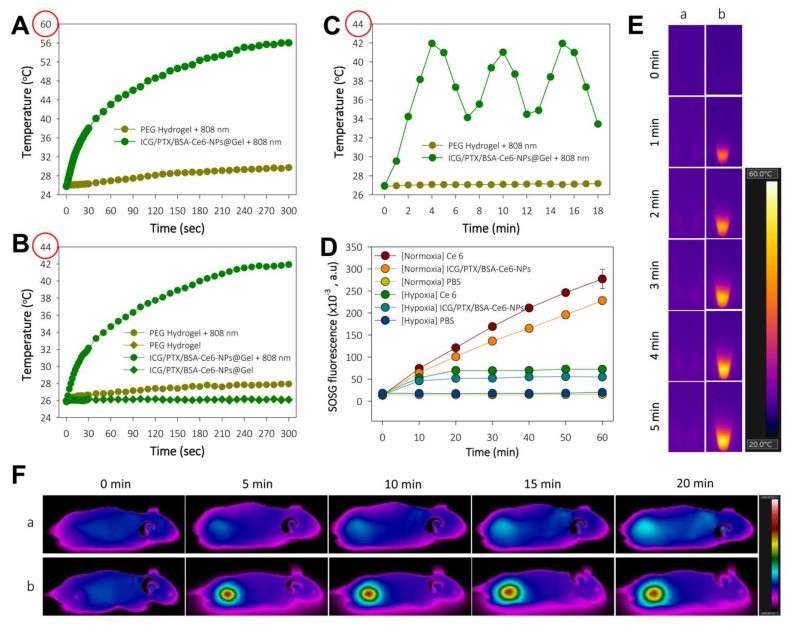
(**A**) Temperature change profile for naïve PEG hydrogel and ICG/PTX/BSA-Ce6-NPs@Gel after targeted 808-nm laser irradiation (1.2 W/cm^2^) to induce severe hyperthermia with. (**B**) Temperature change profile for naïve PEG hydrogel and ICG/PTX/BSA-Ce6-NPs@Gel with or without targeted 808-nm laser irradiation (0.8 W/cm^2^) to induce moderate hyperthermia. (**C**) Temperature adjustment profile for ICG/PTX/BSA-Ce6-NPs@Gel during alternating on and off 808-nm laser irradiation (0.8 W/cm^2^) for 3 min cycles. (**D**) SOSG fluorescence profiles of Ce6 and ICG/PTX/BSA-Ce6-NPs in response to continuous 660 nm-irradiation (10 mW/cm^2^) as assessed by singlet oxygen generation under normoxic and hypoxic conditions. (**E**) Thermal images of (a) naïve PEG hydrogel and (b) ICG/PTX/BSA-Ce6-NPs@Gel during 808 nm laser irradiation (1.2 W/cm^2^). (**F**) In vivo whole-body thermal images of mice bearing 4T1 tumor injected with (a) naïve PEG hydrogel or (b) ICG/PTX/BSA-Ce6-NPs@Gel plus 808-nm laser irradiation (0.8 W/cm^2^) over 20 min.

The induction of hyperthermia by ICG/PTX/BSA-Ce6-NPs@Gel when irradiated by the 808 nm laser was evaluated. As shown in Figure 5A,E, the temperature of the ICG/PTX/BSA-Ce6-NPs@Gel quickly increased from 26 to 56 °C within 5 min of initiation of 808-nm laser irradiation (1.2 W/cm^2^), whereas the temperature of the plain PEG hydrogel increased by only 3 °C. However, the temperature of ICG/PTX/BSA-Ce6-NPs@Gel increased to 42 °C in response to 808 nm laser irradiation (0.8 W/cm^2^) (Figure 5B). As shown in Figure 5C, when 808-nm laser irradiation (0.8 W/cm^2^) was applied intermittently in 3 min on/off cycles, the gel temperature reached ~41 °C after 30 s irradiation and returned to the basal temperature before additional irradiation. Separately, ICG/PTX/BSA-Ce6-NPs were clearly able to induce singlet oxygen generation, as evaluated by the fluorescence intensity caused by SOSG. Overall, the SOSG fluorescence appeared to increase with the duration of irradiation (Figure 5D). Singlet oxygen generation by ICG/PTX/BSA-Ce6-NPs appeared comparable to that of free Ce6, and much less singlet oxygen was generated under hypoxic conditions than under normoxic conditions. Furthermore, the tumor temperature of 4T1 tumor-bearing mouse treated with ICG/PTX/BSA-Ce6-NPs@Gelplus 808-nm laser irradiation (0.8 W/cm^2^) maintained around ~41 °C for 20 min, whereas that of mouse treated with the naïve PEG hydrogel at the same condition did not significantly increase (Figure 5F).

### 3.4. Cytotoxicity Evaluation of 4T1 Cell Spheroids Based on LIVE/DEAD™ Assay

**Figure 6:**
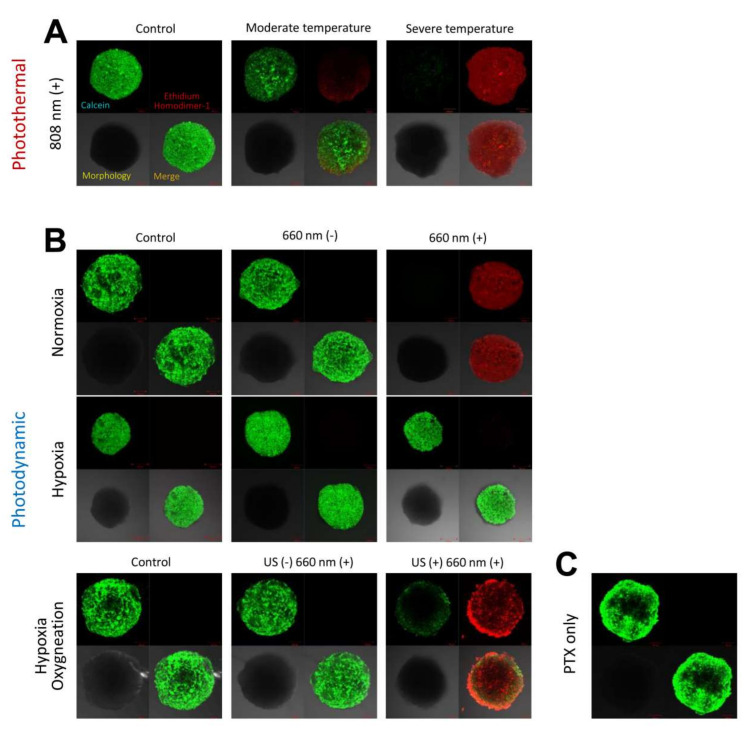
(**A**) CLSM images for 4T1 cell spheroids incubated with ICG/PTX/BSA-Ce6-NPs@Gel under 808 nm laser irradiation specifically adjusted to induce regular, moderate, or severe temperature. (**B**) CLSM images for 4T1 cell spheroids incubated with ICG/PTX/BSA-Ce6-NPs@Gel with or without 660 nm laser irradiation under normoxic, hypoxic, or oxygenated conditions. (**C**) CLSM images for 4T1 cell spheroids incubated with PTX/BSA-NPs@Gel. Green and red colors represent live and dead cells, respectively.

With regard to photothermal effects, when irradiated with the 808 nm laser at 1.2 W/cm^2^ for 10 min, 4T1 cell spheroids appeared to be absolutely dead, showing clear red fluorescence color, whereas 4T1 spheroids were almost all alive after irradiation with the 808 nm laser at 0.8 W/cm^2^ for 10 min, showing mostly green fluorescence color (Figure 6A). With regard to photodynamic effects, the 4T1 cell spheroids cultured with PBS and ICG/PTX/BSA-Ce6-NPs/BSA-PFC-NEs seemed all alive when held under hypoxic conditions, irrespective of 660 nm laser irradiation. However, the 4T1 cell spheroids cultured with ICG/PTX/BSA-Ce6-NPs and irradiated with the 660 nm laser for 60 min were obviously dead when held under normoxic conditions, whereas non-irradiated 4T1 cell spheroids cultured under the same condition showed negligible cytotoxicity. Furthermore, the 4T1 cell spheroids incubated with ICG/PTX/BSA-Ce6-NPs/BSA-PFC-NEs and irradiated with the 660 nm laser (60 min) and also subjected to a US scan (15 min) were mostly dead because oxygen release from the BSA-PFC-NEs was induced by ultrasound scan energy, whereas the 4T1 cell spheroids cultured under the same conditions but without the US scan clearly remained alive (Figure 6B).

### 3.5. Visualization of Hypoxia in 4T1 Cell Spheroids

**Figure 7:**
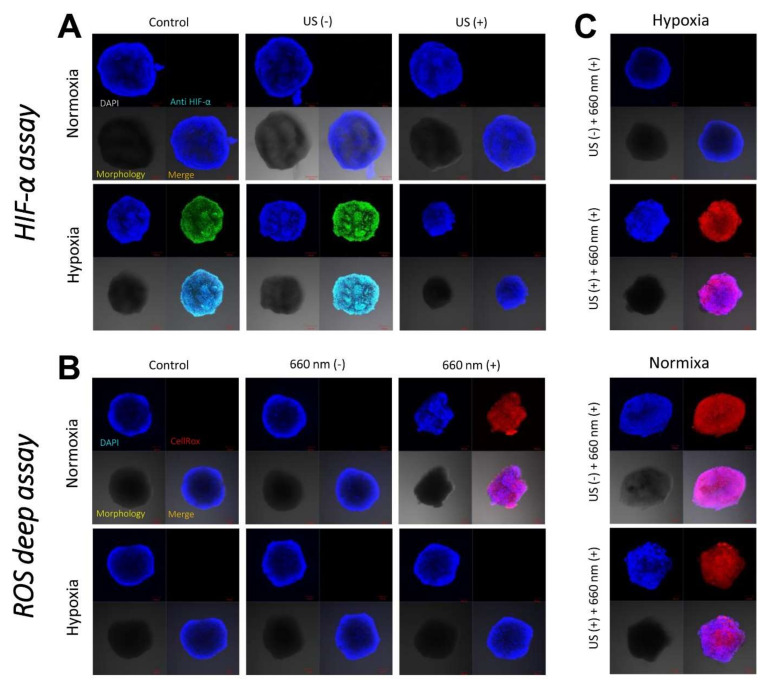
(**A**) CLSM images of 4T1 cell spheroids incubated with ICG/PTX/BSA-Ce6-NPs~PFC-NEs@Gel with or without US guidance under normoxic or hypoxic conditions in order to assess consequent HIF-1α production. (**B**) CLSM images of 4T1 cell spheroids incubated with ICG/PTX/BSA-Ce6-NPs~PFC-NEs@Gel with or without 660 nm laser irradiation under normoxic or hypoxic conditions in order to assess consequent intracellular ROS (singlet oxygen) production. (**C**) CLSM images of 4T1 cell spheroids incubated with ICG/PTX/BSA-Ce6-NPs~PFC-NEs@Gel with or without US guidance and irradiated with a 660 nm laser under normoxic or hypoxic conditions in order to assess consequent intracellular ROS production.

Hypoxic conditions were precisely achieved at the hypoxic chamber in a hypoxic gas stream (5% CO_2_:95% N_2_ on a volumetric basis), and the resulting hypoxia was demonstrated by visualizing the hypoxia-inducible factor (HIF)-1α at 4T1 cell spheroids. As shown in Figure 7A, spheroids treated at hypoxia were strongly stained with anti-HIF-1α antibody, displaying green fluorescence. On the other hand, spheroids treated at normoxia was not stained with anti-HIF-1α antibody because HIF-1α was negligibly expressed. However, 4T1 cell spheroids incubated with ICG/PTX/BSA-Ce6-NPs/BSA-PFC-NEs that were not subjected to a US scan displayed strong green fluorescence when cultured under hypoxic conditions. In contrast, the same 4T1 cell spheroids oxygenated by a US scan did not show the typical hypoxic fluorescence signal.

### 3.6. Visualization of Singlet Oxygen Generation in 4T1 Cell Spheroids

The intracellular ROS levels/distributions were visualized in each group of 4T1 cell spheroids by CLSM. When cultured under hypoxic conditions, all test groups of 4T1 cells incubated with ICG/PTX/BSA-Ce6-NPs~PFC-NEs@Gel did not exhibit strong red signals indicating significant intracellular ROS production, as detected by the ROS-sensitive dye CellROX^®^ Deep Red, regardless of the use of 660 nm laser irradiation (Figure 7B). In contrast, when cultured under normoxic conditions, all 4T1 cell spheroids incubated with the various hydrogels and irradiated with the 660 nm laser displayed strong red fluorescence signals, and this phenomenon appeared even when the spheroids were cultured under hypoxic conditions but oxygenated by a US scan of the PEG hydrogel samples (Figure 7C).

### 3.7. In Vivo Tumor Localization of ICG/PTX/BSA-Ce6-NPs~PFC-NEs@Gel in 4T1 Tumor-Bearing Mice

**Figure 8:**
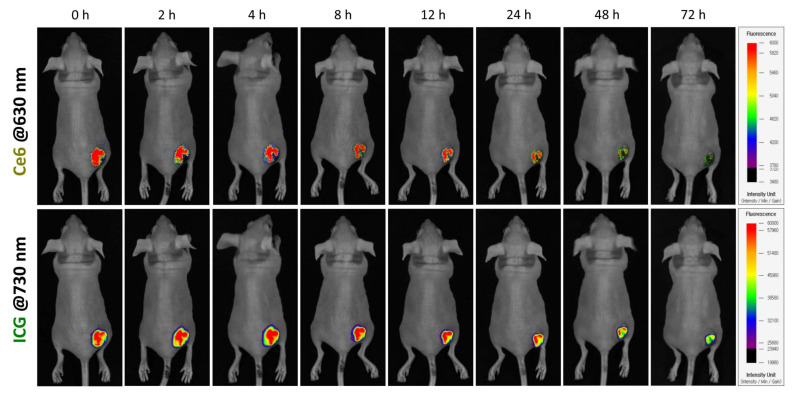
In vivo visualization of 4T1 tumor-bearing mouse after intratumor injection of ICG/PTX/BSA-Ce6-NPs~PFC-NEs@Gel based on Ce6 and ICG fluorescence, at 630 and 730 nm, respectively.

After intratumor injection of ICG/PTX/BSA-Ce6-NPs~PFC-NEs@Gel, ICG and Ce6 fluorescence were visualized in xenografted 4T1 tumor-bearing mice. The ICG and BSA-Ce6 from the PEG hydrogel were diffusely distributed within the tumors. The considerable fluorescence levels were maintained until 12 h, but their NIR-fluorescence signals gradually decreased and then faded out 72 h after intratumor injection (Figure 8).

### 3.8. Photoacoustic Imaging of 4T1 Cell-Xenograft Tumors

**Figure 9:**
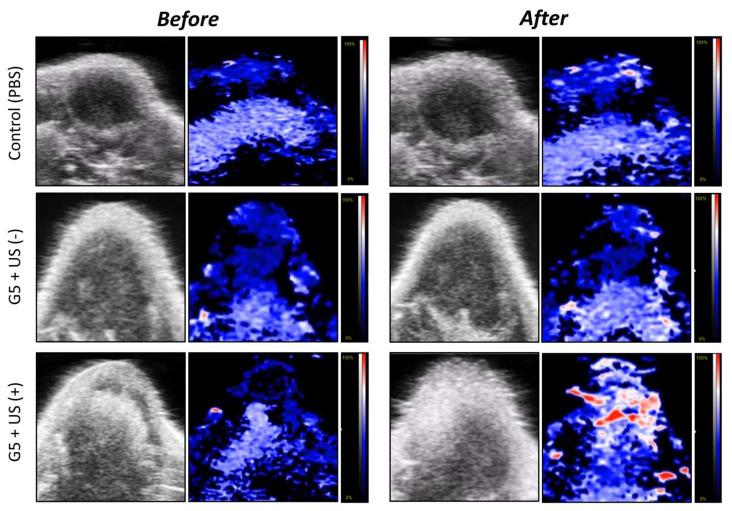
Photoacoustic images of 4T1 cell tumors of mice before and after (at 20 min) intratumor injection of PBS or ICG/PTX/BSA-Ce6-NPs~PFC-NEs@Gel with or without US trigger.

The accumulation of oxygen (as HbO_2_) produced in 4T1 tumors xenografted onto mice was visualized using photoacoustic (PA) analyses with high resolution and deep imaging depth (Figure 9). Groups were designated as either the PBS control or ICG/PTX/BSA-Ce6-NPs~PFC-NEs@Gel (G5 formula) with or without a US scan. The PEG hydrogel group (G5) with US scan under mild heat (41−42 °C) clearly displayed the greatest oxygen distribution (red signal) throughout the tumors on PA imaging: the HbO_2_ percentage was measured as 25.0% to 42.7% at before and after US, respectively, compared with the negligible HbO_2_-level changes (around 23.1%~29.6%) for PBS control and G5 + US(−). This result demonstrated that our ICG/PTX/BSA-Ce6-NPs~PFC-NEs@Gel had a significant ability to supply oxygen to the hypoxic breast tumors.

### 3.9. Antitumor Efficacy in 4T1 Tumor-Bearing Mice

**Figure 10:**
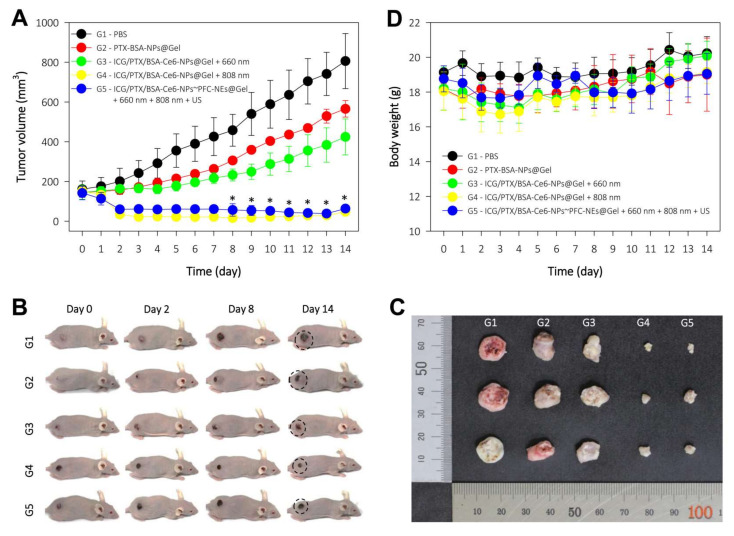
In vivo antitumor efficacy of each PEG hydrogel formulation in five treatment groups of 4T1 tumor-bearing mice (G1−G5): (I) control = PBS; (II) chemotherapeutic = PTX/BSA-NPs@Gel (100 μg PTX (0.1 mL/mouse)); (III) deoxy-photodynamic = ICG/PTX/BSA-Ce6-NPs@Gel + 660 nm (+); (IV) severe hyperthermia = ICG/PTX/BSA-Ce6-NPs@Gel + 808 nm(+); (V) oxy-photodynamic plus moderate hyperthermia = ICG/PTX/BSA-Ce6-NPs@Gel + BSA-PFC-NEs + 660 nm (+) + 808 nm(+) + US(+). All treatments were injected intratumorally. (**A**) Tumor volumes in each group over 14 days. (**B**) Representative photographs of 4T1-tumor-bearing mice at the indicated days after treatment. (**C**) Photographs of tumors excised from each treatment group. (**D**) Body weight change of 4T1-tumor-bearing mice in the different treatment groups over 14 days post treatment.

The antitumor effects of ICG/PTX/BSA-Ce6-NPs~PFC-NEs@Gel were evaluated in 4T1 tumor-bearing mice. The tumor volumes for each group of mice were measured for 14 days after treatment (Figure 10A): the final tumor volumes for the G1 to G5 groups were 805.9 ± 138.5, 565.5 ± 41.6, 424.2 ± 90.6, 47.7 ± 11.6, and 63.4 ± 13.0 mm^3^, respectively. The G4 formulation with severe heat-induced regression of almost the entire tumor in each xenografted mouse due to the hyperthermal effect. In addition, the G5 formulation (with oxygen provided by the US scan and mild heat induced by the 808 nm laser and singlet oxygen generated by 660 nm laser irradiation) noticeably suppressed the tumors as compared to the G3 group that did not receive oxygen induced by the US scan. The body weight of the mice in the five treatment groups was maintained without significant change over 14 days, indicating that all mice were well cared for without deleterious effects during the entire therapy period (Figure 10E).

## 4. Discussion

Combined anticancer treatment using different therapeutic modalities has attracted great attention as an effective way of suppressing malignant tumors [37]. In particular, co-therapy using PDT and PTT may accomplish synergistic efficacy based on different modes of anticancer treatment [26,38]. While PDT is a well-established technique that is commonly used clinically, is minimally invasive, and produces few side effects [39], PTT is used for two distinct purposes in practice: (i) independent treatment to suppress tumors by severe hyperthermia and (ii) supportive treatment to improve the delivery of chemotherapeutics and oxygen into tumors by increasing tumor blood flow due to moderate hyperthermia. Moreover, concurrent use of chemotherapeutics could accelerate tumor ablation [40]. To this end, we focused on enhancing hypoxia-induced tumor suppression by the use of intratumorally injectable PEG hydrogel formulations based on combined effects of chemotherapeutic, photodynamic, and photothermal modalities plus an oxygen-generation system.

For photodynamic therapy, Ce6 was chosen because it is one of the most efficient and safe second-generation PSs that is approved by the FDA for clinical use. It is sensitive to the light of a wide range of wavelengths (red to NIR: 650−900 nm), which penetrates relatively deep into tumors (presumably hypoxic regions) [39]. However, Ce6 is difficult to formulate on account of its poor solubility in both water and chloroform/dichloromethane; hence, albumin conjugates of Ce6 have been used to surmount this difficulty [2,41]. On the other hand, PFC formulated in albumin-stabilized nano-emulsion can be used to potentiate the photodynamic activity of Ce6 in hypoxic tumors by providing oxygen. Several methods have been introduced to supply or generate extra-/intracellular oxygen (i.e., CaO_2_/catalase [7], MnO_2_/H_2_O_2_ [9,42], and a PFC oxygen shuttle/US [35,43]). Among these, PFC is chemically inert and has been explored as an artificial blood substitute due to its high affinity for oxygen. Indocyanine green (ICG) was considered the first option considered for photothermal therapy because it is a deep-penetrating NIR dye approved by the US FDA; in addition, ICG is multifunctional and is also used as a diagnostic and photodynamic agent [44]. However, ICG has several undesirable properties, including concentration-dependent aggregation, poor aqueous stability, and rapid renal elimination from the body, which limit its clinical use [45,46].

The primary aim of this study was to fabricate an in situ-injectable “one-pot” delivery system optimal for including Ce6, ICG, PFC, and PTX in an attempt to achieve concurrent photodynamic, photothermal, and chemotherapeutic effects as a result of improved oxygenation of hypoxic tumors. Previously, we developed a simple method to prepare an in situ-gelling albumin-cross-linked PEG hydrogel system. This hydrogel system comprised only two components (thiolated albumin and 4-arm PEG-maleimide (20 kDa)) and is easily gelled within ~60 s in situ [26,27,36]. If required, the gelation time could be easily adjusted from approximately 15 s to 5 min according to the intended purpose [27]. Likewise, as shown in Figure 3 and Figure 4, all formulations of our ICG/PTX/BSA-Ce6-NPs~PFC-NEs@Gel appeared to be gelled well within 60 s and to have acceptable elastic gel characters: all PEG hydrogel formulations (Z1−Z6) did not flow or drop when held upside-down for 60 s. In clinical settings, this 60 sec period can be critical to allow medical doctors or nurses to complete a series of injection steps: (i) mixing/dissolving powder materials with sterile water for injection, (ii) filling a syringe with the resulting solution, and (iii) immediately injecting the hydrogel into relevant sites before gelation occurs. Likewise, the gelation time for our PEG hydrogels, including those with added ICG/PTX/BSA-Ce6-NPs and BSA-PFC-NEs, was flexibly controlled for 30−120 s depending upon the BSA-SH:4-arm PEG-MAL molar ratio (=1:4.3−4.5). The prepared PEG hydrogel was confirmed to contain ICG/PTX/BSA-Ce6-NPs and BSA-PFC-NEs and displayed moderate to severe hyperthermal and photodynamic effects as measured by a photothermal camera and SOSG fluorescence (Figure 5). In addition, formation of the PEG hydrogel was the result of a specific reaction between artificially driven thiol groups in BSA and maleimide groups of the 4-arm PEG-MAL, and thus the delay or interruption of the release of BSA-Ce6, ICG, or PTX/PFC due to conjugation with PEG frames could be theoretically excluded. Consequently, BSA-Ce6, ICG, and PTX were almost released within 24~36 h (Figure 3B), and oxygen release from BSA-PFC-NEs seemed to be triggered by ultrasound and contributed to the creation of normoxic conditions.

Albumin nanoparticles/nano-emulsions conjugated with Ce6, ICG, and PFC were used as nano-systems inside the PEG hydrogel. Owing to problems with the solubility and stability of Ce6 and ICG, nab^TM^-technique-based albumin nanoparticles were considered to be an optimal formulation to stably load both Ce6 and ICG into the hydrogels. Previously, our group has optimized albumin nanoparticle formulations for Ce6 and ICG [2,30]. Basically, the strongly hydrophobic chemotherapeutic agent PTX played a role in physically cross-linking albumin molecules, and Ce6-conjugated BSA (BSA-Ce6) was embedded within the resulting ICG/PTX/BSA-Ce6-NPs during their preparation. Despite high water solubility, ICG is known to strongly bind to albumin and thus is highly encapsulated into albumin nanoparticles because the aromatic and heteroaromatic rings of ICG interact strongly with hydrophobic pockets within albumin molecules [45,47]. As shown in Figure 2A,C, these two photothermal and photodynamic agents were simultaneously incorporated into albumin nanoparticles of ~175 nm diameter, and the resulting ICG/PTX/BSA-Ce6-NPs were able to provide both activities in response to the laser irradiations (660 and 808 nm). On the other hand, liquid PFC was easily formed through ultrasonication as a nano-emulsion (BSA-PFC-NEs) stabilized by surrounding albumin molecules, showing ~320 nm emulsion size, and thus appeared to be loaded into the PEG hydrogel. Importantly, the entrapped ICG/PTX/BSA-Ce6-NPs and BSA-PFC-NEs appeared not to undergo sudden release from the PEG hydrogel because the hydrogel mesh formed by four-arm PEG (Mn 5−20 kDa) is thought to be ~5 to 15 nm as estimated using the Flory–Rehner equation or rubber elasticity theory [48]. However, ICG and BSA-Ce6 of relatively small size (<6 nm) may be released through the mesh pores and move (in)to target cancer cells.

Our PEG hydrogel formulated with added ICG/PTX/BSA-Ce6-NPs and BSA-PFC-NEs had significant ability to kill 4T1 cell spheroids due to both 808 nm laser-induced hyperthermia and 660 nm laser-induced photodynamic cell death, accompanied by oxygen release from US-triggered BSA-PFC-NEs. Whereas cell death induced by PTX alone seemed insignificant, the production of naïve oxygen and singlet oxygen from our PEG formulations was clearly observed in anti-HIF-1α and ROS deep assays, respectively (Figure 7 and Figure 8). In addition, our ICG/PTX/BSA-Ce6-NPs~PFC-NEs@Gel unambiguously increased oxygen supply to the tumor region as shown in the photoacoustic imaging (Figure 9), and the PEG hydrogel was retained within the area of the injection site for more than three days. This indicated that additional laser irradiation or US scanning might result in much better tumor suppression/regression. Overall, the severe hyperthermal tumor ablation effect of our formula combined with the strengthened photodynamic activity due to oxygenation and moderate hyperthermia (41–42 °C) appeared to potentiate hypoxic tumor ablation in 4T1 tumor-bearing mice.

## 5. Conclusions

In summary, the in-situ injectable PEG hydrogel with added ICG/PTX/BSA-Ce6-NPs (~175 nm) and BSA-PFC-NEs (~320 nm) was easily and quickly (~60 s) fabricated and displayed photothermal, photodynamic, and oxygen-supplying activity. The hypoxic tumors of mice treated with our PEG hydrogel formula and with 808/660 nm laser irradiation plus ultrasound scanning were unambiguously suppressed in volume compared with those of all other groups. This remarkable tumor size reduction is attributable to the strengthened photodynamic activity due to oxygenation and moderate hyperthermia (41~42 °C) by the multifunctional effect of loaded Ce6, ICG, and PFC. In addition, our PEG hydrogel formula had sufficient hyperthermal activity to ablate the 4T1 tumors when the irradiation time and output intensity increased. We believe our multifunctional PEG hydrogel formula can be a prototype for ablating otherwise unresponsive hypoxic tumors in coordination with deep-penetrating outer stimuli.

## Figures and Tables

**Table 1:** Formulations and treatment groups used in this study.

Hydrogel Formulation Groups	Characteristics	Abbreviation	Laser Irradiation	Ultrasound
Z1	PEG hydrogel	-	-	-
Z2	PEG hydrogel with PTX/BSA-NPs	PTX/BSA-NPs@Gel	-	-
Z3	PEG hydrogel with BSA-PFC-NEs	BSA-PFC-NEs@Gel	-	-
Z4	PEG hydrogel with PTX/BSA-Ce6-NPs	PTX/BSA-Ce6-NPs@Gel	-	-
Z5	PEG hydrogel with ICG/PTX-BSA-NPs	ICG/PTX-BSA-NPs@Gel	-	-
Z6	PEG hydrogel with ICG/PTX/BSA-Ce6-NPs and BSA-PFC-NEs	ICG/PTX/BSA-Ce6-NPs~PFC-NEs@Gel	-	-
Animal Group	Intratumor Treatment Groups		Laser Irradiation	Ultrasound
G1	PBS	-	-	-
G2	PEG hydrogel with PTX-BSA-NPs	PTX/BSA-NPs@Gel	-	-
G3	PEG hydrogel with ICG/PTX/BSA-Ce6-NPs	ICG/PTX/BSA-Ce6-NPs@Gel	660 nm (+)	-
G4			808 nm (+)	-
G5	PEG hydrogel with ICG/PTX/BSA-Ce6-NPs and BSA-PFC-NEs	ICG/PTX/BSA-Ce6-NPs~PFC-NEs@Gel	660 nm/808 nm (+)	US (+)

## Data Availability

Not applicable.
